# Amblypygids of Timor-Leste: first records of the order from the country with the description of a remarkable new species of *Sarax* (Arachnida, Amblypygi, Charinidae)

**DOI:** 10.3897/zookeys.820.30139

**Published:** 2019-01-28

**Authors:** Gustavo Silva de Miranda, Ana Sofia P. S. Reboleira

**Affiliations:** 1 Entomology Department, National Museum of Natural History, Smithsonian Institution, Washington DC, USA National Museum of Natural History, Smithsonian Institution Washington United States of America; 2 Natural History Museum of Denmark, University of Copenhagen, Universitetsparken 15, DK-2100 København Ø, Denmark University of Copenhagen Copenhagen Denmark

**Keywords:** cave, tailless whip scorpions, troglobiont, Wallacea

## Abstract

The whip spider genus *Sarax* Simon, 1892 is widely distributed throughout Southeast Asia and part of the Indo-Malayan region. The genus is recorded from several Indonesian islands, but no species are known from inside the area that comprises the biogeographical region of Wallacea, despite being recorded from both sides of the area. An expedition to survey the biology of caves in Timor-Leste (formerly East-Timor) discovered populations of amblypygids living underground and including a remarkable new species of *Sarax*, *S.timorensis***sp. n.**, the first Amblypygi known from the island of Timor. The new species is here described bears the unique character state of two pairs of lateral eyes, instead of three or none as in all other living species of Amblypygi, and expands the biogeographic range of the genus. New records of amblypygids are given for two caves in Timor-Leste. A detailed description and a discussion of its distribution and the species characters are also provided.

## Introduction

Southeast Asia is a global hotspot of biodiversity hitherto poorly known regarding amblypygid diversity. Among the many islands in Southeast Asian seas (Banda, Celebes, Flores, Java, Molucca, Sulu, etc.), only 15 have records of whip spiders. Recent efforts to uncover the amblypygid fauna in that region provided new species of *Catageus* Thorell, 1889 (Charontidae) and *Sarax* Simon, 1892 (Charinidae), and creation of a new genus *Weygoldtia*Miranda et al., 2018 (Miranda et al. 2018; Seiter and Wolff 2017; Seiter et al. 2015).

Four families of Amblypygi are known in Southeast Asia: Charontidae, Charinidae, Phrynidae, and Phrynichidae. Charinidae is the most diverse and has the widest distribution (Miranda et al. 2018). All three genera of the family (*Charinus* Simon, 1892, *Sarax*, and *Weygoldtia*) are present in that region, *Sarax* being the most diverse. *Sarax* species are recognized by the presence of ventral sac cover on the abdomen, presence of a seta on the lateral side of the triad of eyes and female gonopods with finger-like or sucker-like projections (Miranda et al. 2018). The genus occurs in Southeast Asia and the Indo-Malayan region, being recorded from both sides of the Wallacea shelf (on the Sunda and Sahul shelves), but never on islands inside this diverse area.

Timor-Leste (formerly East Timor) is located on the east side of Timor Island in Southeast Asia. Despite long scientific interest in the caves of Timor-Leste for the archeological sciences (O’Connor et al. 2017), almost no information is available on its diversity of subterranean-adapted species (Freire et al. 2017). Moreover, no record of amblypygids was known from the country. Recent intensive collecting in caves around the entire country provided a new view of the subterranean invertebrates of Timor-Leste, including the new species here described.

## Materials and methods

Specimens were collected by active search in caves in Timor-Leste and are deposited in the Natural History Museum of Denmark, University of Copenhagen (NHMD) in 70% ethanol. The material was analyzed under a LEICA M205 stereomicroscope attached to a camera lucida. Photographs were made using a BK plus Imaging System from Visionary Digital (Palmyra, PA, USA; http://www.visionarydigital.com) equipped with a Canon 7D digital camera. Stacks of images from multiple focal planes were combined using Zerene Stacker (Zerene Systems LLC, http://zerenesystems.com/cms/stacker) and processed in Photoshop CS6 (Adobe, San Jose, CA, USA) to adjust color, brightness, and contrast, and remove blemishes. The distribution map was produced using SimpleMappr (www.simplemappr.net).

Nomenclature generally follows Quintero (1981), while the measurements and the terminology of pedipalp and leg segments follows Harvey and West (1998). The article called the tarsus by Harvey and West (1998) is divided here into tarsus and claw, as there is no fusion of these two segments in Charinidae. The spines of the pedipalpal patella and teeth of the chelicerae are counted from the apex to the base. Measuring of the pedipalp articles was taken between the condyles of each segment in order to establish fixed points and comparable length.

Geographically close species, such as *Catageusorientalis* (Seiter & Wolf, 2017), *Charongrayi* (Gervais, 1842), *Charonoenpelli* Harvey & West, 1998, *Charontrebax* Harvey & West, 1998, *Phrynusexsul* Harvey, 2002 and *Saraxbrachydactylus* Simon, 1892 were compared based on the literature (Giupponi and Miranda 2012; Harvey 2002; Harvey and West 1998; Seiter and Wolff 2017; Weygoldt 1996; 2000).

**Other material examined**:

*Charinuspescotti* Dunn, 1949: Australia, Queensland, Freshwater Creek, at Crystal Cascades, 10 km S of Freshwater [17°00'S, 145°40'E], 12.vii.1986, MS Harvey and PJ Vaughan leg. (1 male, 1 subadult male, 1 juvenile, WAM T57789); Flying Fish Point, Innisfail, 21.i.1975, RJ Raven, leg. (1 female, WAM T57787); 2 km WNW of Cape Tribulation, 23.ix–7.x.1982 (1 male, QM S38776); NeS of Pilgrim Sands, Cape Tribulation, 28.viii.1988, R Raven, T Churchill, J Gallon leg., (1 female with eggs, QM S38792); 2 km WNW of Cape Tribulation (site 2), 23.ix–7.x.1982, Monteith, Yeates and Thompson leg. (1 male, 2 females with egg sac, 9 juveniles, QM S38788); West Claudie R. (river), Iron Range, G Monteith and D Cook leg., 3–10.xii.1985, Rainforest, 50 m (2 juveniles, QM S38787)

*Kronocharonlongicalcaris* Wunderlich, 2015: Holotype: Mid Cretaceous Burmite (1 possibly male, F2729/BU/CJW).

*Saraxhuberi* Seiter, Wolff & Horweg, 2015: Paratypes: Republic of Philippines, Cebu: surrounding of the Busay cave [9°54'57.5"N, 123°26'13.2"E], Moalboal, iii.2008, S Huber leg. (1 female, SMNS); South of Moalbaol [9°48'20.1"N, 123°22'17.7"E], Kawasan Falls, 21.ii.2001, S Huber leg. (1 female, 1 male SMNS). Non-type: East slope Mt McKinley, Davao Prov., Mindanao, 26.ix.1946, FG Werner leg., Philippine Zool. Exped. 1946-7, 3300 ft. asl., in tall stump (1 female, 1 juvenile, FMNH 3489489); East slope Mt McKinley, Davao Prov., Mindanao, 10.x.1946, FG Werner leg., Philippine Zool. Exped. 1946-7, 3000 ft. asl., debris on agricultural land (1 male, 1 juvenile, FMNH 3489491); East slope Mt McKinley, Davao Prov., Mindanao, 14.viii.1946, H Hoogstraal leg., Philippine Zool. Exped. 1946-7, 4000 ft. asl., forest floor (1 female, 1 juvenile, FMNH 3489498).

*Saraxsarawakensis* (Thorell, 1888): Indonesia: Bali, Candidasa, Tenagan [8°28'16.2"S, 115°34'8.4"E], 17.iii.2009, S Huber leg. (1 male, AMNH LP 11594); Nusa Tenggara Barat, Sumbawa, Batoedoelang, Sunda Exped., 1927, Rensch leg. (SMF 17.393); no further information (CUMZ I.46520).

*Saraxwilleyi* Gravely, 1915: Papua New Guinea: New Britain: S. küst, Hamburg. Südsee Exped., 5–8.ii.1909, G Duncker leg. (1 male and 6 juveniles, ZMUH); Ralum, 21.x.1896, Dahl L leg. [abdomen detached from the body] (1 female, ZMUH).

*Saraxyayukae* Rahmadi, Harvey & Kojima, 2010: Malaysia: Sarawak: Kuching, Mjóhesg leg., GS Miranda det. (1 female, 1 male, AMNH Ambly 87); Racer Cave [04°03'25.9"N, 114°49'36.8"E], Gunung Mulu National Park, 2–3.viii.2013, 34 m asl., L. Qie, J. Huff, L. Kumpang, M Peter and A Ang leg., GS Miranda det. (3 female, 3 males, 3 juveniles, AMNH LP 12152). Malaysia: Sabah: Pulau Gaya [06°00'44.8"N, 116°00'34.8"E], Tunku Abdul Rahman National Park, 25–28.7.2013, 2 m asl., SF Loria and J Huff leg., GS Miranda det. (5 females, 5 males, 4 juveniles, AMNH LP 12109); Sandakan: British N. Borneo, 8.vii.1929, KP Schmidt leg. (1 male, FMNH 3489490);

*Weygoldtiadavidovi* (Fage, 1946): Syntypes: Vietnam (as Annam): PhanRang, iii–iv.1939, Mission Dawydoff, 1938–39, (3 males, MNHN); Ba Ngoi [11°54'27.23"N, 109°7'21.37"E], x.1938, Mission Dawydoff, 1938–39, Indochine (2 females, plus 1 specimen MNHN); GiaRai (as Giaray) [09°15'36.51"N, 105°22'31.11"E], Bac Liêu, xii.1938–iii.1939, Mission Dawydoff, 1938–39, Indochine (2 males and 3 juveniles, MNHN); Sóc Traeng [09°36'9.01"N, 105°58'25.95"E], Sóc Traeng, xi.1938, Mission Dawydoff, 1938–39, Indochine (1 male, MNHN). Laos: Pak Lay [18°13'50.03"N, 101°24'30.12"E], Xiangnabouli, i.1939, Mission Dawydoff, 1938–39, Indochine (1 juvenile, MNHN). Cambodia: Ream [10°35'2.75"N, 103°38'35.34"E], iii.1939, Mission Dawydoff, 1938–39, Indochine (1 female, 2 juveniles, MNHN).

**Acronyms**:

**AMNH**American Museum of Natural History, New York (Lorenzo Prendini);

**CJW** Collection of Joerg Wunderlich (Joerg Wunderlich);

**CUMZ**Cambridge University Museum of Zoology, UK (Matt Lowe);

**FMNH**The Field Museum of Natural History, Chicago (Petra Sierwald);

**MNHN**Muséum national d’Histoire naturelle, Paris (Mark Judson);

**NHMD** Natural History Museum of Denmark, University of Copenhagen (Nikolaj Scharff);

**QM**Queensland Museum, Brisbane (Robert Raven);

**SMF**Senckenberg Museum für Naturkunde, Frankfurt (Peter Jäger);

**SMNS**Staatliches Museum für Naturkunde, Stuttgart (Joachim Holstein);

**WAM**Western Australian Museum, Perth (Mark Harvey);

**ZMB**Zoologisches Museum Berlin (Jason Dunlop);

**ZMUH**Zoologisches Museum für Hamburg, Hamburg (Matthias Glaubrecht).

## Taxonomy

### Charinidae Quintero, 1986

#### *Sarax* Simon, 1892

##### Sarax timorensis sp. n.

Taxon classificationAnimaliaAmblypygiCharinidae

http://zoobank.org/6E232CB2-B2CC-45A9-9E19-A9BACEE0CBC5

Figs 1 , 2 , 3

###### Type material.

**Holotype**: *Timor-Leste*: Lautém district, Puropoko Cave, 8.543832N 127.066215E, 6–12.ix.2016, A.S.P.S. Reboleira leg. (male, NHMD). Female unknown.

###### Diagnosis.

*Saraxtimorensis* sp. n. can be recognized by the large size (body total length 12.82 mm), presence of only two pairs of lateral eyes, eight frontal setae, cheliceral claw with six teeth, two spines on dorsal pedipalp tarsus, male gonopod with sclerotization on the base of fistula, dorsal lobe and lateral lobe II, basitibia IV with four pseudoarticles and distitibia IV with six trichobothria on the frontal and caudal series. The new species can be distinguished from its congeners by the presence of only two pairs of lateral eyes, a unique character state known only from a few fossil species (*Kronocharonlongicalcaris* Wunderlich, 2015 and *Paracharonopsiscambayensis* Engel & Grimaldi, 2014). *Saraxtimorensis* sp. n. differs from the fossil species by the size (new species much larger) and the number of spines on the pedipalp. Female unknown.

###### Description of holotype male.

**Figure 1.**
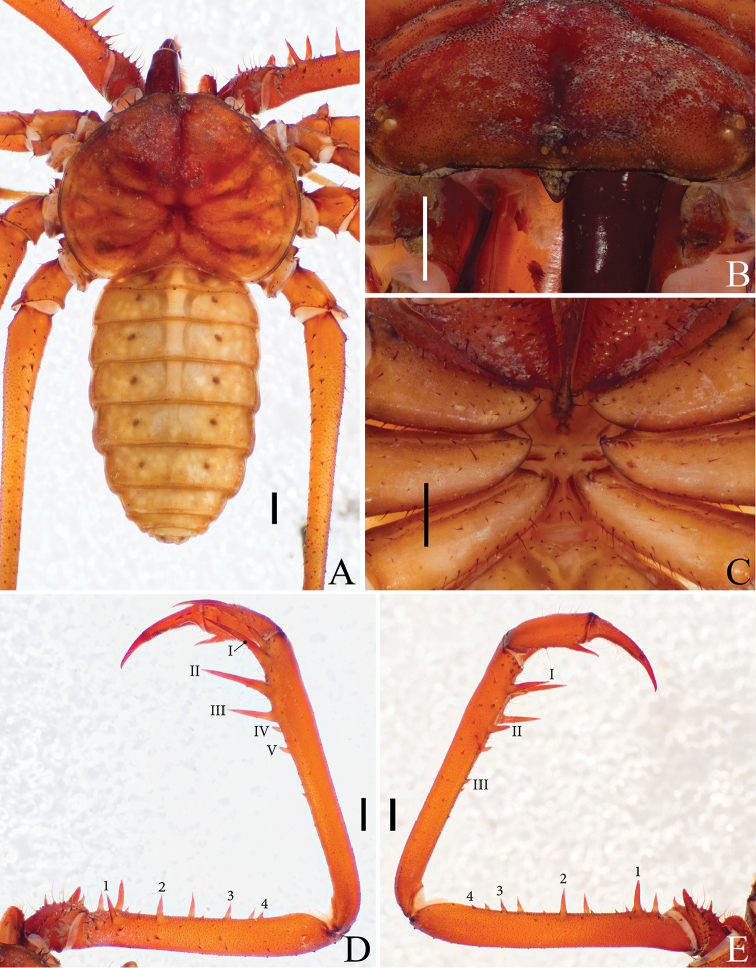
Details of *Saraxtimorensis* sp. n. **A** Dorsal habitus **B** Frontal process and eyes **C** Sternum **D** Dorsal view of pedipalp **E** Ventral view of pedipalp. Scale bar: 1 mm.

**Figure 2.**
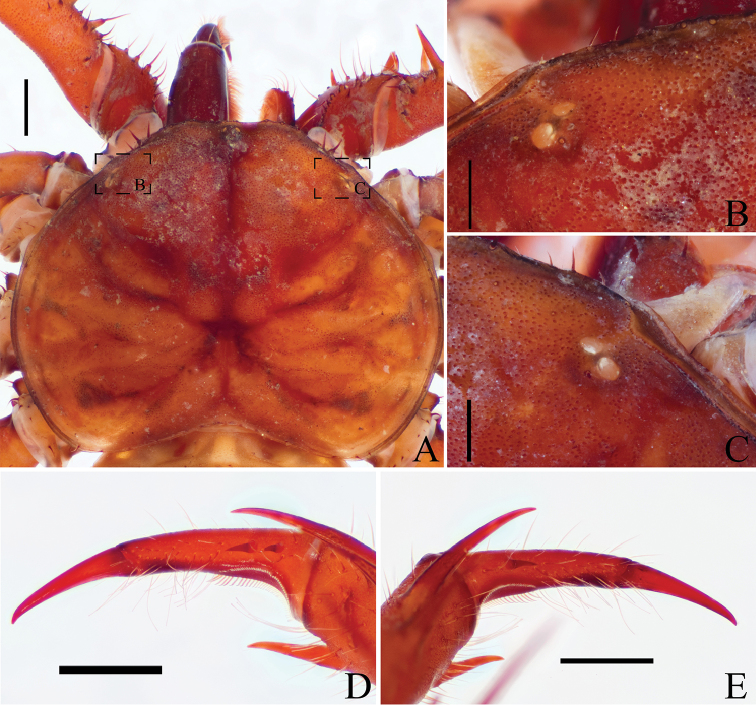
Details of carapace and pedipalp of *Saraxtimorensis* sp. n. **A** Dorsal view of carapace **B** Detail of the left pair of eyes **C** Detail of the right pair of eyes **D** Detail of the spines on right dorsal tarsus **E** Details of spines on left dorsal tarsus. Scale bar: 1 mm (**A, D, E**); 0.5mm (**B, C**).

(All variation are from right-left asymmetry): **Carapace** (Figs 1A, B; 2A–C) with small granules scattered between the lateral eyes and among the sulcs. Median eyes and tubercle weakly developed (Fig. 1B); one pair of setae on the median tubercle; two pairs of lateral eyes (Fig. 2B, C) weakly developed, pale colored, with one setae lateral to the eyes; lateral eyes close to the border of the carapace; presence of a curved crest between lateral eyes and the border of the carapace; eight frontal setae; frontal process well developed, triangular, not seen from above. **Tritosternum** (Fig. 1C) projected anteriorly with the typical Charinidae setation; tritosternum long, surpassing the base of the pedipalp coxae; other sternal platelets narrow and projected, with a pair of setae on the top of the plaque and some smaller ones in the base; pentasternum with four setae close to the membranous region and two setae distally.

**Figure 3.**
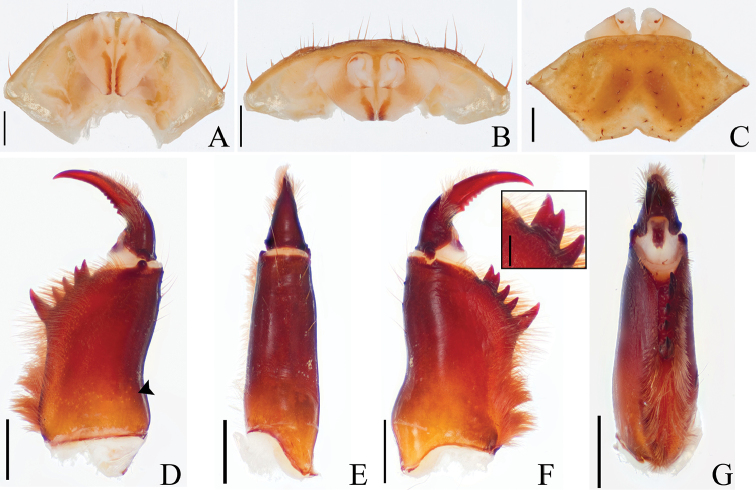
Male gonopod and chelicera of *Saraxtimorensis* sp. n. **A** Dorsal view of male gonopod **B** Posterior view of male gonopod **C** Ventral view of male gonopod **D** Mesal view of right chelicera **E** Dorsal view of right chelicera; detail of the small projection **F** Ectal view of right chelicera **G** Ventral view of right chelicera. Scale bar: 0.5 mm (**A–C**); 1mm (**D–G**); 0.25mm (**F** inset).

**Chelicera** (Figs 3D–G) with a broad and short projection on the ectal side, opposite to the bifid tooth, on the basal segment; ectal side of cheliceral claw (Fig. 3F) with row of setae until the middle of the claw; cheliceral claw with six teeth; mesal side of basal segment proximally with several setae in more than two rows; bifid tooth of basal segment with four teeth in the row with upper cusp larger than lower.

**Abdomen** with ventral sacs cover well developed. Male genital operculum (Figs 3A–C) with short setae in the border of the genital plaque; longer setae scattered over the setae of the genital operculum; inner border of the fistula well sclerotized (Figs 3A, B); base of LoL 2 sclerotized; LoL 2 fimbriated; PI not surpassing the border of the LaM.

**Pedipalp** coxae without setae inside the round carena and with 3–4 setae in its border. Pedipalp trochanter with ventral apophysis pointing forward (Fig. 1D, E), bearing more than 20 strong setae; one spine below the apophysis half the size of the projection of the apophysis and one spine in the middle of the trochanter, in the same row of the long setae. Pedipalp femur (Fig. 1D, E) with four dorsal and with 3–4 ventral spines in the main series; femur dorsal with three prominent setiferous tubercles between the first spine and the proximal margin; femur dorsal with one smaller spine between spines 1–2 and 2–3; femur ventral with one long spine between spine 1 and proximal margin of the segment, two thirds spine 1; femur ventral with 2–3 spines between spines 1–2 and one spine between spines 2–3. Pedipalp patella (Fig. 1D, E) dorsal with 4–5 spines in the main series; one prominent spines distal to spine I; patella ventral with 3–4 spines decreasing in size from distal to proximal; three small setiferous tubercle between spine I and distal border. Pedipalp tibia (Fig. 1D, E) dorsal with two long spines, the distal longer than the proximal; tibia ventral with one distal spine and 5–7 long setae between spine and distal margin. Pedipalp tarsus (Fig. 1D, E; 2 D, E) with two dorsal spines in the right palp and one in the left palp; the two spines are short, subequal; cleaning organ with 37 setae in the ventral row. Tibia I with 23; tarsus I with > 26 articles (both legs incomplete; this number refers to the leg with most of the articles); first tarsal article the same size as the following article. Basitibia IV divided in four pseudo-articles, with a sclerotized, denticulated border in the apex of the articles; *bt* in the distal third of the pseudo-article; distitibia IV with trichobothria *bc* closer to *sbf* than to *bf*, and *sc* and *sf* with six trichobothria each.

**Measurements** (in mm): Carapace: length 4.96, width 6.64. Body total length: 12.82. Pedipalp (right-left): femur 7.76–8.0, patella 7.52–7.62, tibia 2.36–2.60, tarsus 1.88–1.94, tarsal claw 1.16–1.28. Leg I femur 20.8. Leg IV: femur 11.7, basitibia IV–I 9.0, basitibia IV–II 2.6, basitibia IV-III 2.6, basitibia IV–IV 2.6, distitibia 5.25, basitarsus 2.56, other tarsal articles 1.48.

###### Habitat.

The new species was found in a cave on the border of the Ira Lalaro Lake, a huge closed karst depression in the Eastern part of the Timor Island (Freire et al. 2017; O’Connor et al. 2017). The atmospheric temperature in the cave is 32 °C. The cave has a stream with a high density of leeches in its substrate and harbors a large colony of Chiroptera. Some snakes were also observed hunting the bats in its narrow galleries. The high content in bat guano gives rise to high densities of cockroaches which are very active along the cave.

#### Other records

**Figure 4.**
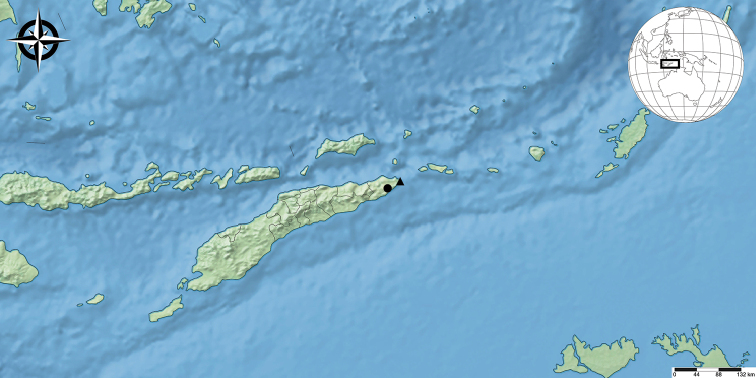
Distribution map of the new records of *Sarax* in Timor-Leste. Key: circle *Saraxtimorensis*, triangle *Sarax* spp.

Two juveniles were found in Acitaukuru Cave (Fig. 4), another cave in the Tutuala forest. These specimens very likely belong to two different species of *Sarax* (apart from *S.timorensis* sp.n.), but they are not described here because no adults were collected. The two specimens were collected in the same cave, but at different depths. Precise localities are:

*Sarax* sp. 1: Timor-Leste: Acitaukuru Cave, -8.415626S, 127.290737E, 6–12.ix.2016, ASPS Reboleira leg. (juvenile, NHMD).

*Sarax* sp. 2: Timor-Leste: Acitaukuru Cave, -8.415626S, 127.290737E, 6–12.ix.2016, ASPS Reboleira leg. (juvenile, NHMD).

## Discussion

Amblypygids of the family Charinidae have, in general, a uniform morphology, but variation is found in several small characters. The number of spines on the pedipalp, for example, varies in distantly related species; the number of teeth on the chelicera also tend to be unique to the different species groups; the same occurs with the position and number of trichobothria on the legs and the number of articles on the first pair of legs and the shape of the female and male genitalia (Giupponi and Miranda 2016; Miranda et al. 2018; Miranda et al. 2016; Weygoldt 2000). Remarkably, some species even lack the median and/or lateral eyes, such as in *C.aguayoi* Moyá-Guzmán, 2009, *C.bichuetteae* Giupponi & Miranda, 2016, *C.bonaldoi* Giupponi & Miranda, 2016, *C.caribensis* (Quintero, 1986), *C.dominicanus* Armas & González, 2001, *C.guto* Giupponi & Miranda, 2016, *C.muchmorei* Armas & Teruel, 1997, *C.reddelli* Miranda, Giupponi & Wizen, 2016, *C.troglobius* Baptista & Giupponi, 2002, and *C.vulgaris* Miranda & Giupponi, 2011, among others. The complete absence of eyes in charinid whip spiders is even rarer and is known only in *Charinusbordoni* (Ravelo, 1977) and *Charinusstygochthobius* Weygoldt & van Damme, 2004. In other amblypygid families, this character state occurs in the paracharontid species *Paracharoncaecus* Hansen, 1921 and the phrynid species *Paraphrynusreddelli* Mullinex, 1979.

*Sarax* species lacking eyes are unknown and there is no record of a living species with a different disposition of ocelli than the usual two median plus three lateral pairs, or no median and three lateral pairs or no eyes at all. A distinct eye arrangement, however, is known in extinct taxa. Some species of the genera *Kronocharon* Engel & Grimaldi, 2014 and *Paracharonopsis* Engel & Grimaldi, 2014 have two pairs of lateral eyes, similar to *S.timorensis* sp. n.; they are *K.longicalcaris* and *P.cambayensis*, respectively. The first is from Burmese amber from ca. 100 million years ago (mya) and belongs to the Unidistitarsata clade; the second is an Indian amber inclusion from ca. 56 mya which is nested within *Paracharon* in Paracharontidae (Engel and Grimaldi 2014; Garwood et al. 2017). The two-lateral-eyes arrangement present in the three species could be a plesiomorphic character state shared among three distinct branches in the Amblypygi tree; another hypothesis is that the species evolved the character state independently as result of pressure from the environment they inhabit or used to inhabit.

Subterranean arthropods tend to have the troglomorphy syndrome, consisting of eye and pigment degeneration, combined with hypertrophy of appendages and sensorial organs, and changes in their metabolism and reproduction. This happens as a result of selective environmental pressures typical of the aphotic and oligotrophic subterranean ecosystem (Culver and Pipan 2014). *Saraxtimorensis* sp. n. does not exhibit highly troglomorphic traits, yet this is one of the largest species in Charinidae, and the tendency to gigantism is also marked in caves as it happens in islands. The largest described species in the family is *Saraxcavernicola* Rahmadi, Harvey & Kojima, 2010 from Indonesia (East Kalimantan), with carapace length ranging between 4.57–6.37 mm and carapace width from 6.30 to 8.29 mm, femur length with 5.95–8.98 mm and patella length measuring 6.15–9.08 mm (Rahmadi et al. 2010). The new species *S.timorensis* becomes the second largest followed by *Weygoldtiadavidovi* (Fage, 1946) from Vietnam. All these species inhabit tropical forests in Southeast Asia. The precise habitat of *K.longicalcaris* and *P.cambayensis* is unknown, but the preservation of the specimens suggest that they lived in a forested area, so troglomorphic pressures likely do not apply in their cases.

The closest geographical amblypygid species to *Saraxtimorensis* sp. n. are *Phrynusexsul* Harvey, 2002 found in caves on Flores Island, Indonesia (Harvey 2002), *Charongrayi* (Gervais, 1842), *Charonoenpelli* Harvey & West, 1998, and *Catageusorientalis* (Seiter & Wolff, 2017) found in the surrounding islands and in Australia (Harvey and West 1998; Seiter and Wolff 2017). The closest *Sarax* species is likely to be *Saraxwilleyi* Gravely, 1915 from West Papua Province in Indonesia (Giupponi and Miranda 2012), located 899.5 km away. Papua-New Guinea and West Papua are part of the Sahul shelf and bear two species of *Sarax*. Almost all other records of the genus are recorded from the Sunda shelf, another Southeast Asian biogeographic region. Nevertheless, the Wallacea shelf, which is the intermediate zone between Asia and Oceania, had no records of the genus so far. The discovery of a *Sarax* species in Wallacea fills a gap in the distribution of the group and poses doubts on the effects of the Lydekker and Wallace’s lines on the distribution of whip spiders in Southeast Asia. Future works will address these biogeographic questions within amblypygids.

Amblypygids in Southeast Asia are still poorly known regarding their systematics, distribution, and biology. Almost 100 records are known for the order in the area and several islands remain without published records for whip spiders. Therefore, it is expected that more species will be found across the region.

## Supplementary Material

XML Treatment for Sarax timorensis
